# Maternal Age of Menarche and Blood Pressure in Adolescence: Evidence from Hong Kong’s “Children of 1997” Birth Cohort

**DOI:** 10.1371/journal.pone.0159855

**Published:** 2016-07-25

**Authors:** Tsz Chun Lai, Gabriel Matthew Leung, C. Mary Schooling

**Affiliations:** 1 School of Public Health, Li Ka Shing Faculty of Medicine, The University of Hong Kong, Hong Kong SAR, People’s Republic of China; 2 City University of New York, School of Public Health and Hunter College, New York, New York, United States of America; Indiana University, UNITED STATES

## Abstract

**Background:**

Age of puberty has declined substantially in developed settings and is now declining in the rest of the world with economic development. Early age of puberty is associated with non-communicable diseases in adulthood, and may be a long-term driver of population health with effects over generations. In a non-Western setting, we examined the association of maternal age of menarche with blood pressure in late childhood/adolescence.

**Methods:**

We used generalised estimating equations to estimate the adjusted association of maternal age of menarche with age-, sex- and height-adjusted blood pressure z-score from 10 to 16 years in Hong Kong’s population-representative birth cohort, “Children of 1997” (n = 8327). We also assessed whether associations were mediated by body mass index (BMI) or pubertal stage.

**Results:**

Earlier maternal age of menarche was associated with higher systolic blood pressure in adolescence [-0.02 z-score per year older maternal age of menarche, 95% confidence interval (CI) -0.04 to -0.003]. The association of maternal age of menarche with systolic blood pressure was mediated by adiposity and/or pubertal stage at 11 years. Maternal age of menarche was not associated with diastolic blood pressure.

**Conclusion:**

Earlier maternal age of puberty was associated with higher systolic blood pressure, largely mediated by adiposity, highlighting the importance of tackling childhood obesity as a public health priority in view of the secular trend of declining age of puberty.

## Introduction

There is a secular trend of decreasing age of onset of puberty globally, which may be stabilising in long-term developed settings but is more marked in rapidly developing settings [1]. Earlier age of puberty, within one generation, is consistently associated with chronic diseases in later life, including the metabolic syndrome [2], atherosclerosis [3], breast cancer [4] and testicular cancer [5], as well as cardiovascular disease risk factors, such as high blood pressure, in childhood and adolescence [3, 6, 7]. Hypertension is a major contributor to the global burden of disease [8], because of its role in cardiovascular disease. Blood pressure tracks from early life [9]. Drivers of blood pressure in children and adolescents may be intervention targets as well as providing etiologic insight concerning targets for cardiovascular disease prevention. Obesity, which may also have an early-life origin [10] and be partially driven by the secular trend of declining age of puberty [5], is a key driver of blood pressure [11, 12].

Whether associations of earlier puberty with non-communicable diseases are limited to one generation, or extend across generations has seldom been examined. All previous studies have found earlier maternal age of menarche associated with higher body mass index (BMI) in childhood [13–16]. A small study found a positive association of maternal age of menarche with systolic blood pressure in girls after 12 years of age [17]. To address this question more fully, we took advantage of a population-representative birth cohort, “Children of 1997”, from the developed non-Western setting of Hong Kong to assess the association of maternal age of menarche, i.e., age of first menstruation, with blood pressure. Given, earlier maternal age of menarche, was associated with higher BMI, in our cohort [16] as well as in other studies [13–15], we also assessed mediation by BMI, and in a complementary analysis mediation by pubertal stage.

## Materials and Methods

### Source of data

“Children of 1997” is a population-representative Chinese birth cohort (n = 8327) in Hong Kong, which has been described in detail elsewhere [18]. The study was initially established to investigate the impact of second-hand smoke exposure on infant health [19]. It covered 88% of all births from 1 April to 31 May 1997. Participants were recruited during their first post-natal visit to one of the 49 Maternal and Child Health Centres (MCHCs) in Hong Kong, where parents of all new-borns are encouraged to bring their infants for free post-natal check-ups and vaccinations until they are 5 years of age. Baseline information, including parental education, some information about parental migration status and birth characteristics (birth-weight, sex, gestational age), was collected using a self-administered questionnaire. Passive follow-up via record linkage was instituted in 2005 [18] to obtain (1) weight and height from birth to 5 years from the MCHCs (96% success); (2) annual measurements of weight and height (grade 1 onwards) and blood pressure (grade 5 onwards) from the Student Health Service, Department of Health, which provides free annual check-ups for all school students; and (3) death records from the Death Registry. In July 2008, Survey I, which focused on family history, including maternal age of menarche, was sent to the families. Additional postal surveys were conducted in 2010–2. Any missing data and discrepancies were reconciled between each wave of data collection.

### Maternal age of menarche

Maternal age of menarche, which was ascertained at mothers’ age ranging from 26 to 59 years, was collected in Survey I. It was originally recorded in 10 categories in complete years: ≤9, 10, 11, 12, 13, 14, 15, 16, 17 and ≥18. To be consistent with previous similar studies [13, 14] and our previous study [16], it was re-categorised into 5 categories: ≤11, 12, 13, 14 and ≥15 years.

### Blood pressure

The outcomes were all measurements of systolic and diastolic blood pressure (SBP and DBP), usually measured every two years from ages 10 to 16 years. Blood pressure was measured on the right arm in a seated position after at least 10 minutes of rest with an age and size appropriate cuff size using a DINAMAP [20]. For systolic and/or diastolic blood pressure higher than 90^th^ percentile for age, blood pressure was measured for a second time after at least 15 minutes of resting using a sphygmomanometer manually; the second reading was recorded. All measurements of SBP and DBP were considered as sex- age- and height-specific z-scores relative to the United States National High Blood Pressure Education Group reference in 2004 [21, 22], to ensure that any associations were not due to differences in these factors between participants.

### Statistical analysis

We compared baseline characteristics by maternal age of menarche using χ^2^ tests. Adjusted associations of maternal age of menarche with blood pressure z-score in adolescence were estimated from generalised estimating equations (GEE) to account for correlation between blood pressure measurements of the same participant [23, 24]. We assessed whether the associations varied by maternal birthplace or sex from the heterogeneity across strata and the significance of the interaction terms (on an additive scale). Maternal age of menarche was also considered as continuous in years to assess the linear trend in order to avoid any bias from misclassification in groups [25].

Models were built sequentially to assess the role of confounding. Confounders were selected as likely common causes of maternal age of menarche and blood pressure [26]. Model 1 assessed the unadjusted association of maternal age of menarche with blood pressure z-score. Model 2 was adjusted for maternal education, household income, highest parental occupation, maternal age, maternal birthplace, sex and age at measurement. BMI and pubertal stage were considered as mediators rather than confounders because they are more likely factors on the pathway from maternal age of menarche to blood pressure at age 10 to 16 years than causes of maternal age of menarche. We assessed mediation by BMI z-score and Tanner stage at 11 years, which was clinically measured by doctors at the Student Health Service clinics, using Pearl’s mediation formula [27] from which we reported direct and indirect effects and the proportion mediated. We used the mediation package (version 4.4.5) in R for this analysis with 5000 bootstrap resamples to obtain 95% CIs. We assessed whether the association of maternal age of menarche and blood pressure at 11 years was mediated by BMI z-score or Tanner stage at 11 years using multivariable linear regression. The association of maternal age of menarche with blood pressure did not vary by BMI z-score at 11 years (p value for SBP = 0.66; p for DBP = 0.98) or Tanner stage at 11 years (p for SBP = 0.15; p for DBP = 0.33).

Given that our birth cohort is population-representative and has comprehensive baseline data concerning the participants’ and families’ characteristics, we used a combination of inverse probability weighting and multiple imputation to handle missing data [28]. Different missingness models were built, based on SEP, maternal age, parity, breastfeeding, sex and MCHC clinic, and the one with lowest Akaike information criterion was chosen. Inverse probability weights were then estimated from this model using logistic regression, to account for potential differences between those who provided maternal age of menarche in Survey I and those who did not. Missing values of factors in the missingness models were multiply imputed 10 times to ensure the sample size was not reduced. We used inverse probability weights in the analyses. We also performed an available case analysis as a sensitivity analysis, i.e. deleting cases with missing data on variables on an analysis-by-analysis basis, for comparison. We also performed the analysis with blood pressure in mmHg and internally generated blood pressure z-scores to ensure that the choice of reference population was not biasing our results. Data were analysed using Stata version 13 (Stata Corp., College station, TX USA) and R version 3.3.0 (R Development Core Team, Vienna, Austria).

### Ethics statement

Since our participants are children, informed consent was obtained from the parents, next of kin, caretakers or guardians (informants) on behalf of the participants by completing the questionnaire at enrolment as approved by The University of Hong Kong Medical Faculty Ethics Committee. Informed written consent for Survey I was obtained from a parent or guardian. Ethical approval for further studies was obtained from the University of Hong Kong-Hospital Authority Hong Kong West Cluster, Joint Institutional Review Board and/or the Ethics Committee of the Department of Health, Government of the Hong Kong SAR as appropriate.

## Results

Of the original 8327 participants, as of 7^th^ January 2016, 29 had permanently withdrawn. At the time of survey I (2008–09), 26 participants had permanently withdrawn, and 365 were not contactable, giving 7936 potential respondents to Survey I. Among them, 3679 responded and 3180 provided maternal age of menarche, of whom 7 were excluded for giving an invalid response. Among these 3172, after excluding another permanent withdrawal, 515 (16.2%) had maternal age of menarche of ≤11 years, 848 (26.7%) 12 years, 788 (24.8%) 13 years, 495 (15.6%) 14 years, and 526 (16.6%) ≥15 years. Most of the participants (85.7%) had at least one blood pressure measurement from 10 to 16 years. On average there were 1.97 measurements from ages 10 to 16 years for each participant.

Table 1 shows that earlier maternal age of menarche was associated with higher education, higher household income and higher parental occupation. Mothers who were born in the rest of China or elsewhere had later age of menarche. Maternal age of menarche was not associated with maternal age.

**Table 1:** Baseline characteristics by maternal age of menarche from Hong Kong’s “Children of 1997” birth cohort.

		Maternal age of menarche (in complete years), %					
Characteristics	n	≤11(n = 515)	12(n = 848)	13(n = 788)	14(n = 495)	≥15(n = 526)	p-value
Child's sex							0.19
Girl	1711	54.8	53.1	54.2	50.1	57.6	
Boy	1462	45.2	46.9	45.8	49.9	42.4	
Maternal education							<0.001
Grade 9 or below	1195	28.0	28.4	32.9	43.4	64.1	
Grade 10–11	1443	54.4	51.4	49.0	40.0	27.1	
Grade 12 or above	533	17.7	20.2	18.2	16.6	8.8	
Household income per head at birth in quintiles (mean ± SD)							<0.001
1st quintile (HK$ 1751 ± 413)	505	11.3	12.9	14.9	20.7	34.8	
2nd quintile (HK$ 2856 ± 325)	538	15.6	14.6	17.3	22.1	29.8	
3rd quintile (HK$ 4362 ± 556)	555	21.5	19.0	21.5	18.9	17.3	
4th quintile (HK$ 6822 ± 886)	610	26.5	25.9	23.2	19.6	9.3	
5th quintile (HK$ 14850 ± 16050)	610	25.2	27.6	23.1	18.9	8.9	
Highest parental occupation at birth							<0.001
I (professional)	752	30.8	33.2	28.4	22.4	15.2	
II (managerial)	413	16.8	15.4	15.2	14.2	12.2	
IIINM (nonmanual skilled)	812	29.9	29.6	29.4	32.6	24.7	
IIIM (manual skilled)	444	10.9	12.1	15.2	17.5	27.9	
IV (semi-skilled)	275	9.2	8.4	9.6	9.9	13.8	
V (unskilled)	78	2.4	1.2	2.3	3.5	6.1	
Maternal birthplace							<0.001
Rest of China or elsewhere	1205	21.6	24.2	35.3	45.2	74.3	
Hong Kong	1958	78.4	75.8	64.7	54.8	25.7	
Maternal age at birth							0.06
≤24 years	294	12.2	9.2	7.9	7.5	10.3	
25–29 years	980	30.3	28.5	29.7	33.9	34.4	
30–34 years	1269	36.9	42.0	42.0	40.6	36.5	
≥35 years	627	20.6	20.3	20.4	18.0	18.7	

*Numbers may not add up to 100% due to rounding

The association of maternal age of menarche with blood pressure did not vary by sex (p for SBP = 0.48; p for DBP = 0.43) or maternal birthplace (p for SBP = 0.89, p for DBP = 0.92). Table 2 shows that younger maternal age of menarche was not associated with SBP in Model 1. However, after adjustment for maternal education, household income, highest parental occupation, maternal age, maternal birthplace, sex and age at measurement (Model 2) younger maternal age of menarche was associated with higher SBP. Maternal age of menarche was not associated with DBP at puberty in any model. The association of maternal age of menarche with SBP was partially mediated by BMI z-score at 11 years (63.3%) or by Tanner stage at 11 years (41.8%) (Table 3).

**Table 2:** Adjusted association of maternal age of menarche with blood pressure z-score (with reference to CDC Growth Chart) in adolescence (from 10 to 16 years) in the “Children of 1997” Birth Cohort from Hong Kong.

			Maternal age of menarche (years)											
			≤11		12		13		14		≥15			
	Model	n	β	95%CI	β	95%CI	β	95%CI	β	95%CI	β	95%CI	β for trend	95%CI
Systolic blood pressure	1	2977	Ref.	-	-0.05	-0.14 to 0.04	-0.05	-0.14 to 0.05	-0.04	-0.14 to 0.07	-0.11	-0.22 to -0.01	-0.01	-0.03 to 0.01
	2	2977	Ref.	-	-0.05	-0.14 to 0.04	-0.05	-0.15 to 0.04	-0.07	-0.18 to 0.03	-0.16	-0.27 to -0.05	-0.02	-0.04 to -0.00
Diastolic blood pressure	1	2977	Ref.	-	0.00	-0.04 to 0.05	0.00	-0.05 to 0.05	0.00	-0.05 to 0.06	0.03	-0.02 to 0.08	0.01	-0.00 to 0.02
	2	2977	Ref.	-	0.00	-0.05 to 0.05	-0.01	-0.06 to 0.04	-0.02	-0.07 to 0.03	-0.01	-0.07 to 0.05	-0.00	-0.01 to 0.01

Model 1 is the crude model.

Model 2 adjusted for maternal age, maternal education, maternal birthplace, highest parental occupation, household income, sex and age at measurement.β-coefficients represent the change in blood pressure z-score (1-unit change in SBP z-score is approximately 10.6 mm Hg and 1-unit change in DBP z-score is approximately 11.3 mm Hg).

**Table 3:** Total, direct, and indirect effects of maternal age of menarche and 95% CI on systolic and diastolic blood pressure z-score at 11 years with the proportion mediated by BMI z-score and Tanner stage at 11 years.

*Mediation by BMI z-score*							
	**Total effect**		**Direct effect**		**Indirect effect**		**Proportion mediated**
	**β**	**95%CI**	**β**	**95%CI**	**β**	**95%CI**	
Systolic blood pressure	-0.012	-0.032 to 0.008	-0.005	-0.025 to 0.015	-0.008	-0.011 to -0.004	0.633
Diastolic blood pressure	0.000	-0.013 to 0.010	0.000	-0.012 to 0.012	-0.001	-0.002 to 0.000	N/A
*Mediation by Tanner stage*							
	**Total effect**		**Direct effect**		**Indirect effect**		**Proportion mediated**
	**β**	**95%CI**	**β**	**95%CI**	**β**	**95%CI**	
Systolic blood pressure	-0.012	-0.032 to 0.008	-0.007	-0.027 to 0.013	-0.005	-0.008 to -0.002	0.418
Diastolic blood pressure	-0.001	-0.013 to 0.010	-0.001	-0.012 to 0.011	0.000	-0.002 to 0.001	N/A

Models adjusted for maternal age, maternal education, maternal birthplace, highest parental occupation, household income, sex and age at measurement.

Sensitivity analyses using blood pressure in mmHg gave similar results (S1 Table), with younger maternal age of menarche associated with higher SBP (Model 2) but not with DBP. Results were also similar using internally generated blood pressure z-score (S2 Table) and in available case analysis (S3 Table). An available case analysis using blood pressure in mmHg also showed that older maternal age of menarche was associated with lower SBP, but was not associated with DBP (S4 Table).

## Discussion

In this large, prospective, population-representative birth cohort from an under-studied non-Western setting, we found a graded association of maternal age of menarche with SBP in late childhood/adolescence, which was largely explained by adiposity and/or earlier pubertal timing. Maternal age of menarche was not associated with DBP. Our study adds by demonstrating an inter-generational association of earlier puberty with blood pressure, possibly partially driven by the association of earlier maternal age of menarche with greater adiposity and/or with earlier pubertal timing.

In this population-representative study, blood pressure and anthropometric measurements were taken regularly by trained nurses. Nonetheless our study has several limitations. First, maternal age of menarche was self-reported. Age of menarche is a watershed event with good recall years later [29]. Non-differential recall error usually biases towards the null. Second, we have missing data on exposures and some confounders. We used a combination of inverse probability weighting and multiple imputation to handle the missing data, because it is more difficult to fulfil the assumptions required for available case analysis than analysis of imputed data. The combination of inverse probability weighting and multiple imputation allowed us to capitalise on the data we have and increased efficiency [28]. Third, children with lower SEP were less likely to be followed up. However, we included parental education in the inverse probability weights. Fourth, blood pressure was measured with oscillometric devices, or rechecked by sphygmomanometer if the reading exceeded the 90^th^ percentile. Oscillometric devices have slight differences from the gold standard mercury sphygmomanometer [30, 31]. However our analysis is unlikely to be biased differentially by these differences. Fifth, a reference population from the US was chosen for converting blood pressure into z-scores, since there is no published reference for Hong Kong adolescents. The choice of reference population is unlikely to affect the internal comparisons made here, results using internally generated z-scores were similar (S2 Table).

Despite the associations of earlier puberty with many chronic diseases, associations with blood pressure have been inconsistent across settings [32]. For instance, two previous studies, one from the US [33] and the other from China [34], found age of menarche was not associated with blood pressure in later life. In a British cohort, the association was only evident in men [35]. Where an association of earlier puberty with blood pressure was observed, adiposity did not fully explain the association in adolescence [3, 36] or mid-life [2, 37]. However, in the present study, the intergenerational association of earlier puberty with higher blood pressure in adolescence was partially mediated by adiposity and/or pubertal stage. Differences in intergenerational associations for blood pressure and adiposity suggest that blood pressure and adiposity have to some extent different drivers. As such, declining age of puberty over generations [38] might drive obesity more than blood pressure. However, an association could emerge in adulthood. A previous small study found maternal age of menarche associated with higher SBP only in a subset of girls, which might be a chance finding on stratification [17], however, the study is too small to be definitive. To our knowledge, this is the first study investigating intergenerational associations of timing of puberty with blood pressure outside a Western setting, as well as assessing the mediating role of adiposity. At a general level our findings are consistent with the disassociation of secular trends in obesity and blood pressure [39, 40], because maternal pubertal timing might be more relevant to childhood obesity than blood pressure. A secular trend of increasing obesity without a parallel trend in blood pressure has occurred in Western settings for adults [41] and children [40, 42], and also in Asia [43] and other rapidly developing settings [44].

Our finding that an association of earlier maternal age of menarche with higher systolic blood pressure was partially mediated by BMI could indicate that maternal age of menarche operates largely by a mechanism that affects obesity but does not extend to blood pressure. This pattern of associations could have arisen for a number of reasons. First, as well as the association of earlier menarche with cardiovascular risk, obesity also appears to promote earlier puberty [45], whilst blood pressure does not, making a relation of maternal age of menarche with blood pressure less likely. Shared genetic architecture could drive both age of menarche and obesity, but not blood pressure. Genome-wide association studies (GWAS) show common genetic variants, including ADCY3-PDMC and PXMP3, [46] drive both age of menarche and adiposity. In contrast, a shared genetic basis for timing of puberty and blood pressure has not yet been found, although GWAS has to date has only explained a small percentage of the variance in blood pressure (<1%), for reasons that are unclear [47]. Finally, we assessed blood pressure in late childhood and early adolescence. The factors underlying the relation of maternal age of menarche with offspring characteristics may have greater impact at a stage when the drivers of growth affect adiposity more than blood pressure. Alternatively, earlier maternal age of menarche may be associated with a lifestyle that protects more against high blood pressure than obesity, for example a plentiful but low salt diet.

## Conclusions

In a recently developed, non-Western setting, earlier maternal age of menarche was associated with higher systolic blood pressure in late childhood/adolescence, but the association was partially explained by adiposity and/or earlier pubertal timing, suggesting that the association of falling age of menarche with blood pressure, or possibly other non-communicable diseases in adulthood, might be partially driven by adiposity. Our study highlights the importance of tackling childhood obesity as a public health strategy to reduce population cardiovascular risk.

## Supporting Information

S1 Table Adjusted association of maternal age of menarche with blood pressure in adolescence (from 10 to 16 years) in the “Children of 1997” Birth Cohort from Hong Kong. (DOCX)

S2 Table Adjusted association of maternal age of menarche with internal blood pressure z-score in adolescence (from 10 to 16 years) in the “Children of 1997” Birth Cohort from Hong Kong. (DOCX)

S3 Table Adjusted association of maternal age of menarche with blood pressure z-score (with reference to CDC Growth Chart) in adolescence (from 10 to 16 years) in the “Children of 1997” Birth Cohort from Hong Kong (complete case analysis). (DOCX)

S4 Table Adjusted association of maternal age of menarche with blood pressure in adolescence (from 10 to 16 years) in the “Children of 1997” Birth Cohort from Hong Kong (complete case analysis). (DOCX)

## Abbreviations

SBP: Systolic blood pressure
DBP: Diastolic blood pressure
GEE: Generalised estimated equations
MCHCs: Maternal and Child Health Centres
SEP: Socioeconomic position
