# Supplementary material for: Maternal Age of Menarche and Blood Pressure in Adolescence: Evidence from Hong Kong’s “Children of 1997” Birth Cohort
Source: PLoS One. 2016 Jul 25;11(7):e0159855. doi: 10.1371/journal.pone.0159855 (PMC4959736; doi:10.1371/journal.pone.0159855)
Supplement: S1 Table — (DOCX) [file pone.0159855.s001.docx]

**S1 Table**: Adjusted association of maternal age of menarche with blood pressure in adolescence (from 10 to 16 years) in the “Children of 1997” Birth Cohort from Hong Kong

|  |  |  | **Maternal age of menarche (years)** | | | | | | | | | |  |  |
| --- | --- | --- | --- | --- | --- | --- | --- | --- | --- | --- | --- | --- | --- | --- |
|  |  |  | **≤11** |  | **12** |  | **13** |  | **14** |  | **≥15** |  |  |  |
|  | **Model** | **n** | **β** | **95%CI** | **β** | **95%CI** | **β** | **95%CI** | **β** | **95%CI** | **β** | **95%CI** | **β for trend** | **95%CI** |
|  |  |  |  |  |  |  |  |  |  |  |  |  |  |  |
| Systolic blood pressure | 1 | 2977 | Ref. | - | -0.57 | -1.63 to 0.48 | -0.70 | -1.79 to 0.39 | -0.60 | -1.81 to 0.61 | -1.68 | -2.87 to -0.50 | -0.23 | -0.45 to -0.02 |
|  | 2 | 2977 | Ref. | - | -0.71 | -1.73 to 0.30 | -0.82 | -1.86 to 0.23 | -1.27 | -2.44 to -0.11 | -2.21 | -3.43 to -0.98 | -0.35 | -0.58 to -0.12 |
|  |  |  |  |  |  |  |  |  |  |  |  |  |  |  |
| Diastolic blood pressure | 1 | 2977 | Ref. | - | 0.02 | -0.52 to 0.57 | -0.11 | -0.67 to 0.45 | -0.11 | -0.75 to 0.52 | 0.15 | -0.46 to 0.77 | 0.03 | -0.08 to 0.14 |
|  | 2 | 2977 | Ref. | - | -0.06 | -0.60 to 0.49 | -0.24 | -0.80 to 0.32 | -0.47 | -1.11 to 0.16 | -0.40 | -1.07 to 0.26 | -0.09 | -0.21 to 0.03 |

Model 1 is the crude model.
Model 2 adjusted for sex, age at measurement, maternal age, maternal education, maternal birthplace, highest parental occupation and household income.
β-coefficients represent the change in blood pressure (in mmHg).
