# Supplementary material for: Maternal Age of Menarche and Blood Pressure in Adolescence: Evidence from Hong Kong’s “Children of 1997” Birth Cohort
Source: PLoS One. 2016 Jul 25;11(7):e0159855. doi: 10.1371/journal.pone.0159855 (PMC4959736; doi:10.1371/journal.pone.0159855)
Supplement: S2 Table — (DOCX) [file pone.0159855.s002.docx]

**S2 Table:** Adjusted association of maternal age of menarche with internal blood pressure z-score in adolescence (from 10 to 16 years) in the “Children of 1997” Birth Cohort from Hong Kong

|  |  |  | **Maternal age of menarche (years)** | | | | | | | | | |  |  |
| --- | --- | --- | --- | --- | --- | --- | --- | --- | --- | --- | --- | --- | --- | --- |
|  |  |  | **≤11** |  | **12** |  | **13** |  | **14** |  | **≥15** |  |  |  |
|  | **Model** | **n** | **β** | **95%CI** | **β** | **95%CI** | **β** | **95%CI** | **β** | **95%CI** | **β** | **95%CI** | **β for trend** | **95%CI** |
|  |  |  |  |  |  |  |  |  |  |  |  |  |  |  |
| Systolic blood pressure | 1 | 2977 | Ref. | - | -0.05 | -0.14 to 0.04 | -0.03 | -0.12 to 0.07 | -0.03 | -0.13 to 0.07 | -0.09 | -0.19 to 0.01 | -0.01 | -0.02 to 0.01 |
|  | 2 | 2977 | Ref. | - | -0.04 | -0.14 to 0.05 | -0.03 | -0.12 to 0.06 | -0.04 | -0.15 to 0.06 | -0.13 | -0.24 to -0.02 | -0.02 | -0.03 to 0.01 |
|  |  |  |  |  |  |  |  |  |  |  |  |  |  |  |
| Diastolic blood pressure | 1 | 2977 | Ref. | - | 0.01 | -0.08 to 0.10 | 0.00 | -0.09 to 0.09 | 0.00 | -0.10 to 0.10 | 0.06 | -0.04 to 0.16 | 0.01 | -0.00 to 0.03 |
|  | 2 | 2977 | Ref. | - | 0.01 | -0.08 to 0.09 | -0.01 | -0.10 to 0.08 | -0.03 | -0.14 to 0.07 | -0.02 | -0.13 to 0.09 | -0.00 | -0.02 to 0.02 |

Model 1 is the crude model.
Model 2 adjusted for sex, age at measurement, maternal age, maternal education, maternal birthplace, highest parental occupation and household income.
β-coefficients represent the change in blood pressure z-score (1-unit change in SBP z-score is approximately 10.3 mm Hg and 1-unit change in DBP z-score is approximately 5.6 mm Hg)
