# Supplementary material for: Maternal Age of Menarche and Blood Pressure in Adolescence: Evidence from Hong Kong’s “Children of 1997” Birth Cohort
Source: PLoS One. 2016 Jul 25;11(7):e0159855. doi: 10.1371/journal.pone.0159855 (PMC4959736; doi:10.1371/journal.pone.0159855)
Supplement: S4 Table — (DOCX) [file pone.0159855.s004.docx]

**S4 Table**: Adjusted association of maternal age of menarche with blood pressure in adolescence (from 10 to 16 years) in the “Children of 1997” Birth Cohort from Hong Kong (available case analysis)

|  |  |  | **Maternal age of menarche (years)** | | | | | | | | | |  |  |
| --- | --- | --- | --- | --- | --- | --- | --- | --- | --- | --- | --- | --- | --- | --- |
|  |  |  | **≤11** |  | **12** |  | **13** |  | **14** |  | **≥15** |  |  |  |
|  | **Model** | **n** | **β** | **95%CI** | **β** | **95%CI** | **β** | **95%CI** | **β** | **95%CI** | **β** | **95%CI** | **β for trend** | **95%CI** |
|  |  |  |  |  |  |  |  |  |  |  |  |  |  |  |
| Systolic blood pressure | 1 | 2977 | Ref. | - | -0.58 | -1.62 to 0.47 | -0.74 | -1.82 to 0.34 | -0.64 | -1.84 to 0.55 | -1.68 | -2.86 to -0.51 | -0.23 | -0.45 to -0.02 |
|  | 2 | 2407 | Ref. | - | -0.39 | -1.49 to 0.72 | -0.88 | -2.02 to 0.25 | -1.33 | -2.61 to -0.05 | -1.94 | -3.29 to -0.59 | -0.34 | -0.60 to -0.09 |
|  |  |  |  |  |  |  |  |  |  |  |  |  |  |  |
| Diastolic blood pressure | 1 | 2977 | Ref. | - | 0.00 | -0.55 to 0.55 | -0.12 | -0.68 to 0.43 | -0.15 | -0.78 to 0.49 | 0.14 | -0.47 to 0.76 | 0.03 | -0.08 to 0.13 |
|  | 2 | 2407 | Ref. | - | -0.04 | -0.64 to 0.55 | -0.19 | -0.81 to 0.43 | -0.46 | -1.17 to 0.25 | -0.27 | -1.02 to 0.49 | -0.06 | -0.20 to 0.07 |

Model 1 is the crude model.
Model 2 adjusted for sex, age at measurement, maternal age, maternal education, maternal birthplace, highest parental occupation and household income.
β-coefficients represent the change in blood pressure (in mmHg).
